# Is Tit-for-Tat the Answer? On the Conclusions Drawn from Axelrod's Tournaments

**DOI:** 10.1371/journal.pone.0134128

**Published:** 2015-07-30

**Authors:** Amnon Rapoport, Darryl A. Seale, Andrew M. Colman

**Affiliations:** 1 School of Business Administration, Anderson Hall, 900 University Avenue, University of California, Riverside, California 92521–0203, United States of America; 2 Lee Business School, University of Nevada, University of Nevada Las Vegas, 4505 Maryland Parkway—Box 456009, Las Vegas, Nevada 89154–6009, United States of America; 3 School of Psychology, University of Leicester, Leicester LE1 7RH, United Kingdom; Universidad Carlos III de Madrid, SPAIN

## Abstract

Axelrod’s celebrated Prisoner’s Dilemma computer tournaments, published in the early 1980s, were designed to find effective ways of acting in everyday interactions with the strategic properties of the iterated Prisoner’s Dilemma game. The winner of both tournaments was tit-for-tat, a program that cooperates on the first round and then, on every subsequent round, copies the co-player’s choice from the previous round. This has been interpreted as evidence that tit-for-tat is an effective general-purpose strategy. By re-analyzing data from the first tournament and some more recent data, we provide new results suggesting that the efficacy of tit-for-tat is contingent on the design of the tournament, the criterion used to determine success, and the particular values chosen for the Prisoner’s Dilemma payoff matrix. We argue that this places in doubt the generality of the results and the policy implications drawn from them.

## Introduction

In 1979 Robert Axelrod invited scientists from several different academic disciplines to enter a Prisoner’s Dilemma (PD) round-robin computer tournament. Expressing dissatisfaction with previous research on the iterated PD game that had—in his judgment—failed to reveal how to play the game well, Axelrod argued that a new approach was needed to “learn more about how to choose effectively in an iterated Prisoner’s Dilemma” [1], p. 6., [2], p. 29. For this purpose, he invited 14 scientists, all with previous records of studying the PD, to submit computer programs for participation in a single-stage round-robin tournament involving exactly 200 repeated games against each of the other programs entered into the tournament. The 2 × 2 PD payoff matrix that was used in the tournament had the “conventional values” [3] shown in Table 1. Using standard labeling of payoffs (*T* for sole defection, *R* for joint cooperation, *P* for joint defection, and *S* for sole cooperation), the values in this payoff matrix are (*T*, *R*, *P*, *S*) = (5, 3, 1, 0).

**Table 1:** Payoff Matrix for the PD Game: “Conventional” Values.

	Cooperate (*C*)	Defect (*D*)
**Cooperate (*C*)**	3, 3	0, 5
**Defect (*D*)**	5, 0	1, 1

*Note*. *T* = 5, *R* = 3, *P* = 1, *S* = 0

Each computer program was supposed to embody a set of rules specifying either a cooperative (*C*) or a non-cooperative/defecting (*D*) pure strategy on each repetition of the game. A useful benchmark for very good performance relative to the scoring rule specified by Axelrod is 600 points, equal to the score attained by each player if both always cooperate. A second useful benchmark is 200 points, attained by each if both always defect. As announced in the official rules of the tournament, which were commonly known by all the participants, each entry was also paired with a copy of itself and with another program, called RANDOM, that on each move cooperates or defects randomly with equal probability.

Axelrod believed that the results of a computer tournament might help to discover the best strategy for everyday human interactions with the general strategic structure of PD. The first sentence of the article in which he presented his results was: “This article is a ‘primer’ on how to play the Prisoner’s Dilemma game effectively” [1], p. 3. He argued that the approach he proposed had to take account of two facts about strategic interaction in an iterated non-zero-sum setting. The first is that the effectiveness of any strategy is likely to depend not only on the characteristics of that particular strategy, but also on the nature of the strategies against which it competes. A second and related fact is that an effective strategy must be able to take account of the entire history of the dyadic interaction as it has developed from the outset.

The rules of the tournament instructed contestants to maximize the number of points won across all 15 dyadic interactions (including the interactions with a copy of itself and with an additional program that chose *C* or *D* randomly with equal probability). At the end of the tournament, the competing programs were to be ranked in terms of the total number of points that each had accumulated. The element of dyadic competition in the tournament was thereby suppressed, if not completely eliminated, as nothing was said in the instructions about winning any particular dyadic interaction. One may argue that this is an odd way to determine the overall winner of the tournament. Soccer teams are not evaluated at the end of the season by the number of goals they have scored; chess players are not ranked by the number of pieces they capture from all the rivals; and NBA basketball teams are not ranked by the total number of points they have scored by the end of the tournament.

### Tit-for-tat

It is by now well known that tit-for-tat (TFT), the simplest of the 14 genuine programs submitted (leaving aside the program that merely randomized its choices), ended up amassing the most points and thereby winning the tournament. To remind the reader, TFT chooses the cooperative strategy *C* on move *t* = 1, and on each subsequent move *t* (*t* = 2,…, *n*) it mimics the co-player’s decision at move *t–* 1. TFT carries a memory of the immediately preceding outcome only; it forgets the earlier history of the interaction entirely and plays each move as if it were the last. In contrast to the requirement mentioned above, that an effective strategy should take account of the entire history of the dyadic interaction, it ignores the history apart from the outcome of the previous move, and it cannot signal its intentions or shape the future interaction, except in a very limited sense. Furthermore, an important property of TFT is that it can never win any particular iterated PD game—it can never achieve a positive point difference against any other program. All of this should have been known to the participants, as TFT had been studied earlier by Anatol Rapoport and Al Chammah [4] in their classical book on the PD, and also by Stuart Oskamp [5], Amnon Rapoport [6], and others.

Noting that the effectiveness of any particular program for playing the iterated PD game depends not only on its own characteristics, but also on the characteristics of all other competing programs, Axelrod [7] conducted a second tournament. Entrants were informed of the outcome of the first tournament and the concepts used by Axelrod [1] to explain the reasons for the success or failure of the different programs. The rules for the second tournament were the same as for the first tournament, with the sole exception that the number of repetitions of the game in each pairing, rather than being fixed at 200, was determined probabilistically to minimize end-game effects. A total of 63 programs (including RANDOM) competed in the second tournament. Once again, TFT emerged as the overall winner. Axelrod found the results of the tournaments surprising, and so did the authors of many subsequent articles and books that commented on these two tournaments and their implications.

The computer tournaments have attracted a vast amount of attention and are regarded by many as classic studies. They have been discussed by Axelrod [1], [2], [7], Hofstadter [8], Maynard Smith [9], Anatol Rapoport [10], Selten and Hammerstein [11], Beer [12], Bendor [13], Nowak and Sigmund [14], and Colman [15], among many others. No attempt is being made here to survey the already voluminous and still rapidly growing literature in this area of research, spanning psychology, economics, political science, biology, computer science, and system studies (see, e.g., [3], [16]). Axelrod [17] has commented recently that “the rate of citations for the early work has not yet peaked even after thirty years” (p. 22). Most researchers and commentators seem to have accepted his conclusions regarding the reasons for the robust success of TFT and the policy implications that he suggested for “how to choose successfully” [16]. Axelrod [2] summarized his conclusions as follows:

What accounts for TIT FOR TAT’s robust success is its combination of being nice, retaliatory, forgiving, and clear. Its niceness prevents it from getting into unnecessary trouble. Its retaliation discourages the other side from persisting whenever defection is tried. Its forgiveness helps restore mutual cooperation. And its clarity makes it intelligible to the other player, thereby eliciting long-term cooperation. (p. 54)

Cautionary notes have been sounded by Hofstadter [8] and Colman [15], and subsequent PD computer tournaments reported by Bendor, Kramer, and Stout [18], Donninger [19], and Nowak and Sigmund [14] have not supported TFT as the overall winner. However, to the best of our knowledge, previous researchers have not pointed out that the success of TFT may be contingent on a particular combination of the format chosen for the tournament, the objective function that defined overall success, and the values of the PD payoff matrix used in the tournaments. Our main argument is that the generalizations inferred from the two tournaments, and in particular the policy implications drawn from their results, may not be warranted without unambiguous qualification.

## Tournament Design

Tournaments are competitions that involve relatively large numbers of contestants who participate in a series of games in order to determine the overall winner or, more generally, rank-order the contestants in terms of their performance. In designing a tournament, independent decisions have to be made about three major issues: the format of the tournament, the objective criterion to be maximized, and the population of the contestants.

Alternative formats have been designed and implemented in sports and games. One popular format is the *knockout tournament*, in which the competition is divided into several stages, and each contestant plays at least one fixture at each stage, with the top-ranked competitors in each stage progressing to the next. As the tournament continues, the number of competitors decreases. The winner of the final stage, which consists of a single fixture, is the overall winner. In a *round-robin tournament*, each contestant competes with each of the others an equal number of times, once in a single-stage round-robin tournament and twice in a two-stage round-robin tournament. For example, in the English Premier League, twenty teams compete in multiple soccer matches. Each team is matched with each of the other 19 teams twice, once at its home stadium and once at the opponent’s, for a total of 38 matches for each team. A FIFA World Cup (soccer) tournament combines these two most popular formats in a particular order. The top 32 teams in the 2014 World Cup were first divided into eight groups of four that participated in single round-robin tournaments. Then, the two top teams in each group progressed to the second stage in which the resulting 16 teams participated in a single-elimination (knockout) stage. The World Chess Championship Candidates Tournament in 2014 was an eight-player, two-stage round-robin tournament in which each player faced every other player once as White and once as Black, the winner earning the right to a head-to-head title match with the existing world champion.

### Criteria for success

The criteria for determining the overall winner also vary considerably. Chess tournaments use a simple scoring rule: one point for a win, half a point for a draw, and zero points for a loss. The English Premier League imposes a more complicated scoring system that was introduced to discourage ties. Teams are awarded three points for a win, one point for a tie, and zero points for a loss. At the end of the season, teams are ranked by three criteria that are applied in a lexicographic order: teams are first ranked by the number of points; if these are equal, then ties are broken by the goal difference; and if these goal differences are also equal, then ties are broken by the number of goals scored. In most other tournaments, it is simply the number of wins that counts. In general (e.g., in basketball, American football, and backgammon) point differentials play no role in determining the overall winner. Many tournaments in track-and-field events, soccer, basketball, backgammon, and tennis have qualifying competitions for ensuring that only the most successful players or teams take part in the tournament proper.

Both of the original PD computer tournaments made use of the single-stage round-robin format. In theory, this type of tournament provides a fair procedure for rank-ordering contestants or choosing overall winners. Its primary disadvantage is that it becomes impractical when the number of contestants is large. Clearly, this problem disappears when the tournament is conducted on a computer. The objective criterion chosen for the PD tournaments was maximization of the total number of points across all pairings. Table 2 presents the total number of points won by each program against itself and against each of the others in the first tournament. The 15 competing programs are presented in a descending order of score total, and it is clear from the table that TFT ranked top and RANDOM ranked bottom. The programs are numbered from 1 to 15 in the column labeled “Rank Point.” The next column (second from right), labeled “No. of Wins,” lists the number of wins counted across the 15 pairings, and the last column labeled “Rank Wins” displays the rank-ordering of the 15 entries in terms of number of wins. Our reason for including numbers of wins and the associated rank-ordering is that most tournaments use the number of wins to determine the winner. Although TFT cannot ever win an individual encounter, most of the other programs can. The data in the two right-hand columns are discussed later.

**Table 2:** Tournament Scores in Axelrod’s First Tournament.

Prog.	TFT	T&C	NY	GR	SH	S&R	FR	DA	GR	DO	FE	JO	TU	NA	RAN	Mean	Rank Point	No. of Wins	Rank Wins
**TFT**	600	595	600	600	600	595	600	600	597	597	280	225	279	359	441	**504**	**1**	**0**	**15**
**T&C**	600	596	600	601	600	596	600	600	310	601	271	213	291	455	573	**500**	**2**	**11**	**2**
**NY**	600	595	600	600	600	595	600	600	433	158	354	374	347	368	464	**486**	**3**	**1**	**13.5**
**GR**	600	595	600	600	600	594	600	600	376	309	289	236	305	426	507	**482**	**4**	**4**	**6**
**SH**	600	595	600	600	600	595	600	600	348	271	274	272	265	448	543	**481**	**5**	**3**	**11.5**
**S&R**	600	596	600	602	600	596	600	600	319	200	252	249	280	480	592	**478**	**6**	**10**	**3.5**
**FR**	600	595	600	600	600	595	600	600	307	207	235	213	263	489	598	**473**	**7**	**6**	**8**
**DA**	600	595	600	600	600	595	600	600	307	194	238	247	253	450	598	**472**	**8**	**4**	**9.5**
**GR**	597	305	462	375	348	314	302	302	588	625	268	238	274	466	548	**401**	**9**	**5**	**9.5**
**DO**	597	591	398	289	261	215	202	239	555	202	436	540	243	487	604	**391**	**10**	**6**	**6**
**FE**	285	271	426	286	297	255	235	239	274	704	246	236	272	420	467	**328**	**11**	**12**	**3.5**
**JO**	230	214	409	237	286	254	213	252	244	634	236	224	273	390	469	**304**	**12**	**10**	**1**
**TU**	284	287	415	293	318	271	243	229	278	193	271	260	273	426	478	**301**	**13**	**6**	**6**
**NA**	362	231	397	273	230	149	133	173	187	133	317	366	345	413	526	**282**	**14**	**2**	**11.5**
**RAN**	442	142	407	313	219	141	108	137	189	102	360	416	419	300	450	**276**	**15**	**1**	**13.5**

Why was the criterion of maximizing total number of points across all pairings chosen in Axelrod’s tournaments? Examination of the program descriptions suggests that most of them were not, in fact, designed to maximize the total number of points; rather, they were constructed to win any particular round of play. On the face of it, the criterion might have been chosen in an attempt to foster cooperation—a player would not like to get locked into a sequence of *D*-*D* outcomes and end up with a relatively low score. Evidence in support of this conjecture is that the top-ranking entries in Axelrod’s first tournament were “nice” (defined as never being the first to defect). In fact, the ranking of the eight “nice” programs relative to one another was largely determined by just two of the other “kingmaker” programs that are not nice, namely DO (Downing) and GR (Graaskamp). The concept of kingmaker strategies was introduced by Axelrod [1], pp. 10–13.

The participants in the first tournament were recruited from among “experts” who had written on game theory and, in particular, on the PD game. In the second tournament, entrants were provided with a detailed analysis of the first tournament, including the results presented in Table 2, together with concepts used to analyze success and pitfalls that were discovered. Axelrod [7] remarked: “Therefore, the second round presumably began at a much higher level of sophistication than the first round, and its results should therefore be much more valuable as a guide to effective choice in the Prisoner’s Dilemma” (p. 381).

## Evaluation

Can the conclusions about the superiority of TFT drawn by Axelrod, and further propagated in subsequent papers and books, be generalized beyond the design of his two tournaments? To answer this question, we examined separately the effects of changes in format, objective criterion, and payoff values on tournament outcomes. To study the effects of the format, we chose among formats that are not susceptible to the presence of “kingmakers,” while still controlling in part for the element of luck. In the first analysis, we divided the 15 entries from the original tournament randomly into three groups of five entries, each of which participated in a two-stage round-robin tournament based on the number of points listed in Table 2. In particular, the five entries in each group participated in a single-stage (preliminary) round-robin tournament. The (three) winners, one of each group, then progressed to the second stage in which they participated in a second (final) round-robin tournament to determine the overall winner. An advantage of the two-stage tournament is that it allows “experts” to emerge endogenously on the basis of the outcomes of the first round of the tournament.

### Results of re-analysis

To illustrate the effect of a change in format on the tournament outcome, we repeated this procedure twice (we performed two runs). In the first run, the three groups randomly chosen from Table 2 included the entries ranked {1, 2, 7, 9, 10}, {5, 6, 12, 14, 15}, and {3, 4, 8, 11, 13}. Programs 1, 6, and 3, the winners of their respective groups, proceeded to Stage 2, which was won by Program 6. (Programs 1 and 3 came very close behind within 1 point difference.) In the second run, the three randomly chosen groups included the programs {4, 5, 7, 10, 13}, {2, 3, 9, 11, 15}, and {1, 6, 8, 12, 14}. Programs 4, 3, and 6, the winners of their respective groups in Stage 1, progressed to Stage 2 where once again Program 6 emerged as the winner. As the results of these two examples are by no means representative, we computed all combinations resulting from dividing the 15 programs into three groups of five entries each (a total of 756,756 combinations), and subjected each combination to a two-stage round-robin tournament using the scores in Table 2. Ties that occurred in either the preliminary or the final tournament were broken randomly. The results are summarized in Table 3. The first column in Table 3 lists the programs from 1 to 15, as in Axelrod’s original paper, and the second column lists their names. The third column presents the percentage of times that each program entered the final stage of the tournament, and the fourth column presents the percentage of the two-stage tournaments won by each of the 15 programs.

**Table 3:** Analysis of Axelrod’s First Tournament as a Two-stage Round-Robin Tournament.

Program	Name	% Preliminary	% Wins
1	TFT	42.1	11.0
2	T&C	41.4	30.0
3	NY	41.5	10.2
4	GR	31.0	10.0
5	SH	24.4	6.7
6	S&R	39.9	24.8
7	FR	21.1	4.9
8	DA	13.8	2.1
9	GR	11.8	0.2
10	DO	20.8	0.1
11	FE	7.7	0
12	JO	2.5	0
13	TU	0.4	0
14	NA	1.2	0
15	RAND	0.5	0
		300.0%	100.0%

Table 3 shows that Program 2 (T&C) won 30.0% of the tournaments, Program 6 (S&R) came second with 24.8%, whereas Program 1 (TFT) only won 11.0%. Program 1 was followed closely by Programs 3 and 4 that won 10.2% and 10.0% of all the tournaments, respectively. The top eight programs, all of them characterized as “nice” by Axelrod, accounted for 99.7% of the wins. Table 3 also shows that Program 1 (TFT) won a higher percentage of the preliminary stage (42.1%) than any other program. However, when competing with two other programs in the second and final stage, both of them “nice,” it won only in about 26% of its interactions (in comparison, Program 2 won in almost 75% of its interactions in the final stage). This result confirms Axelrod’s observation that the success of TFT in his first tournament is largely due to two “kingmakers” included in the seven bottom programs in Tables 2 and 3.

To complete this analysis, we matched the top eight entries, all of them “nice,” in a single-stage round-robin tournament. The overall winner among the top eight “nice” programs was Program 6, followed by Programs 2 and 3. TFT was one of the four programs that tied for the lowest ranking. The point of these analyses is to demonstrate that the format of the tournament is critical, and consequently that the conclusions drawn from one format may not be readily generalizable to another.

To readers familiar with tournaments, it may seem obvious that maximizing the total number of points in a single-stage round-robin tournament may not yield the same ranking of contestants as maximizing the number of wins across all dyadic interactions. However, it might be assumed that the rankings yielded by these two separate criteria are likely to be positively and indeed highly correlated. For example, this seems to be the case in the English Premier League: teams that score many goals tend in general to do well in the final ranking. Therefore, to check whether one ranking may serve as a proxy for the other in the PD tournaments, we re-analyzed the results of the first tournament in terms of the number of wins. We recorded a win whenever the winning margin against a co-player was positive; we deleted every game between a program and its twin; and in every case of a tie, we recorded the mean rank. Thus, for example, Table 2 shows that Program FE was ranked 11th in terms of mean number of points won (328) but was ranked first in terms of the number of wins (12). TFT did not score even a single win—by its nature, it can never outscore its co-player—and was ranked last. The Spearman rank correlation between the two rankings turns out to be ρ = *−*.103. The null hypothesis that the two rankings in Axelrod’s first tournament are uncorrelated cannot be rejected (*p >* .70).

We performed a similar analysis using five groups of three programs (rather than three groups of five) and obtained largely similar results. The details of this and a more complicated replication are set out in the supporting information file “S1 Alternative Tournament Formats”. Data and results for the tournament with three groups of five programs are provided as supporting information in the Excel file “S1 Tournament Results”.

### More recent data

One might possibly have expected the two objective criteria to be positively but only imperfectly correlated. But our finding that they are not even positively correlated may come as a surprise. To further assess the generality of this finding, we searched for other tournaments between computer programs playing iterated PD games with possibly different rules and larger numbers of participants. We found a suitable round-robin tournament that was organized in 2004 and its results reported by Kendall, Yao, and Chong [20]. It also used the PD game with the “conventional” payoffs presented in Table 1. In contrast to the tournament organized by Axelrod in 1979, the 2004 tournament incorporated random noise, which resulted in occasional misimplementation of moves. Additionally, competitors could submit multiple programs, and many did so. Altogether, the 2004 tournament included 223 programs. The results are provided in an online table [21], where the number of pairwise competitions won by each program and the sum of points won against all of its co-players are listed. A simple computation reveals that, for *n* = 223, the Spearman rank correlation between the two sets of scores (the number of pairwise interactions won and the total sum of the number of points won) is ρ = *−*.45; it is negative and highly significant (*p <* 0.001).

### Payoff Values

A final comment about the two original PD tournaments concerns the payoff values shown in Table 1. There are infinitely many payoff matrices that satisfy the defining conditions of the PD game (*T* > *R* > *P* > *S* and 2*R* > *P* + *S*), but only one of them was chosen for the original tournaments. Do the particular payoff values matter as long as the matrix satisfies the PD conditions? Anatol Rapoport and Al Chammah [4] provided evidence that the particular values do indeed matter, and it follows that conclusions about cooperation in the PD game may not be readily generalizable from one set of payoffs to another. Rapoport and Chammah compared behavior in seven different variants of the PD game (p. 37) using a design that systematically manipulated the payoff values. The levels of cooperation that they observed varied from 26.8% to 77.4%. Table 4 reproduces one of their seven games (*T*, *R*, *P*, *S*) = (50, 1, –1, –50), the one that elicited the lowest percentage of cooperative choices. If we were to replace the –50 payoff with –200 and the 50 with 200 (to magnify the effect of defection still further), and if we repeated the single round-robin tournament with this new payoff matrix (still a well-defined PD game), would TFT be the overall winner? Considering that a single defection following a long sequence of mutual cooperative choices would wipe out TFT’s cumulative gains and result in a very large point difference, we conjecture that it might not.

**Table 4:** Payoff Matrix for a Low-Cooperation PD Game.

	Cooperate (*C*)	Defect (*D*)
**Cooperate (*C*)**	1, 1	–50, 50
**Defect (*D*)**	50, –50	–1, –1

*Note*. *T* = 50, *R* = 1, *P* = –1, *S* = –50

Evidence relevant to this conjecture comes from Kretz [22], who conducted a long series of computer simulations of an iterated PD single-stage round-robin tournament to investigate the effects of number of iterations, memory size carried by each of the players, and—most relevant to the present paper—values in the 2 × 2 payoff matrix. He summarized his results as follows: “The main result of the tournament as carried out here is that different strategies emerge as winners for different payoff matrices” (p. 384). A more general conclusion that supports our argument about the iterated PD is that conclusions drawn from computational investigations of the iterated PD game may be valid only if they do not depend significantly on the particular values in the payoff matrix.

## Conclusions

The Prisoner’s Dilemma was originally introduced as a non-cooperative two-person game (see, e.g., Luce and Raiffa [23]). Most of the theoretical and experimental literature has studied the game in its original context. It was Axelrod who has shifted the focus by embedding the two-person game in a round-robin tournament in which each program is pitted against the “the field” with the explicit purpose “To learn more about how to choose effectively in an iterated Prisoner’s Dilemma” ([1], p. 6). *We note that Axelrod has been careful not to explicate the notion of “effectiveness”*: *is it maximization of individual payoff*, *maximization of joint payoff*, *maximization of the difference between yours and your opponent’s payoff*, *reaching some pre-determined payoff target*, *or some combination of the above*? It would seem reasonable to interpret “effectiveness” as maximization of individual expected utility (represented by payoffs) in any repeated interaction with a given co-player. This expected utility should not depend on the outcomes of interactions between any other pairs of players who are not involved in the same interaction. However, the criterion used by Axelrod to rank the strategies takes into account payoffs earned by *other* pairs of players who are not involved in the same interaction. Recall that the success of TFT in his tournaments was determined largely by the outcomes of the pairwise interactions of others, in particular those involving the two “kingmakers” (Axelrod’s own term). Axelrod either overlooked this anomaly or preferred to ignore it by not defining “effectiveness” explicitly.

We agree that Axelrod’s “new approach” has been extremely successful and immensely influential in casting light on the conflict between an individual and the collective rationality reflected in the choices of a population whose members are unknown and its size unspecified, thereby opening a new avenue of research. Our purpose is not to detract from this important contribution. Rather, what has motivated our project is the observation that once the two-person PD game is embedded in a tournament, the overall success of each player—however measured—is not only determined by the decisions she and her opponent makes in each stage of the dyadic interaction but also by the decisions of other dyadic interactions in the population. Therefore, decisions have to be made about the format of the tournament, the criteria for determining a “winner”, and the payoff structure. To the best of our knowledge, Axelrod has provided no justification for his choices of the format, criterion for determining “success”, and payoff structure. In an attempt to further extend his “new approach”, we argue that other choices are equally reasonable. We then show that all of his choices matter and, consequently, the policy recommendations about the effectiveness of TFT should be qualified.

Our focus in this article is on the usefulness of round-robin computer tournaments for determining the most effective strategies in interactions with the strategic structure of the PD game. We recognize and appreciate other approaches to evaluating PD strategies, including evolutionary game theory using mathematical analysis (e.g., [3], [24], [25]) or agent-based computer simulation (e.g., [26], [27], [28]), but discussion of such approaches is clearly beyond the scope of this article.

For more than thirty years, in hundreds of publications, social and behavioral scientists have propagated the conclusion that TFT is the appropriate strategy to follow in resolving conflicts in dyadic interactions that satisfy the assumptions underlying the iterated two-person PD game. For example, Jurišić et al. [16], after reviewing the relevant literature up to 2012, concluded: “Prisoner’s dilemma is still a current research area with nearly 15000 papers during the past two years (Source: Google Scholar). New strategies are developed and old ones are reused in new areas. But basic rules for cooperation that were recognized by Axelrod in the first competition are still valid” (p. 1097). Evidence for this conclusion and support for the associated recommendation rest on the outcomes of two round-robin computer tournaments reported by Axelrod [1], [2], [7] and a few additional tournaments with the same format and criterion of success. With one exception that we know of [29], these additional tournaments also followed Axelrod by using the same 2 × 2 payoff matrix from his original tournaments.

One may argue that any strategy proposed to resolve iterative dyadic conflicts of the PD type that calls for ignoring the entire history of the interaction apart from the immediately preceding outcome demands close scrutiny. Our findings challenge the generality of Axelrod’s results and, in particular, their non-critical interpretation by showing that they are restricted to a particular combination of tournament format, criterion for success, and the PD payoff values. We show that other strategies turn out to be most successful when the format, criterion, and PD payoff values differ from those used in the original tournaments. This, in turn, suggests that Axelrod’s original question about how to choose effectively in the iterated PD game is yet to be answered.

## Supporting Information

S1 Alternative Tournament Formats (PDF)

S1 Tournament Results (XLSX)
