# Supplementary material for: Is Tit-for-Tat the Answer? On the Conclusions Drawn from Axelrod's Tournaments
Source: PLoS One. 2015 Jul 30;10(7):e0134128. doi: 10.1371/journal.pone.0134128 (PMC4520619; doi:10.1371/journal.pone.0134128)
Supplement: S1 Alternative Tournament Formats — (PDF) [file pone.0134128.s001.pdf]

# Alternative Tournament Formats

Three alternative tournament formats are described below. The selection of these formats is limited to those using the pairwise scoring, which was previously reported. Specifically, we consider (*i*) a round-robin tournament with three groups of five entries, (*ii*) a second round-robin tournament with five groups of three entries, and (*iii*) a four-stage, single-elimination match-up tournament.

# Round-robin Tournament with Three Groups of Five Entries

This tournament format considered all combinations of the fifteen original strategies placed into three groups, each with five entries. With computer simulation we examined all $\frac{15!}{5!\,5!\,5!} =$ 756,756 tournament combinations. Using the tournament scores reported in Table 2 for each entry we compute the total points earned in the first-stage, round-robin competition with four other entries. The entry with the most points (ties broken randomly) in each group moves on to the second (final) stage where each of the three group winners competes to determine the overall winner. The number of times each entry won the first and second stage was computed over all possible tournament combinations. The results of this simulation, which are reported in Table 3 and discussed in the body of the manuscript, are not repeated here.

# Round-robin Tournament with Five Groups of Three Entries

Using five groups, each with three entries results in $\frac{15!}{3!\,3!\,3!\,3!\,3!}$ = 168,168,000 unique tournament combinations. Examining this many combinations is tedious, even with computer simulation. Thus, we elected to produce a random sample of 100,000 tournament combinations. Similar to the three groups of five tournament format described above, the entry with the most points in each group in the first stage moved on to the second and final stage. As before, ties are broken randomly. Table S1a below shows the program number, name, and number of times the program won the first and final rounds in this round-robin tournament. Consistent with the results reported in Table 3 for the round-robin tournament with three groups of five entries, T&C won

the most tournaments, posting wins in 24.3% of all tournament combinations. While the top eight entries from previous analyses remained in the top eight, there were differences in the percentage of wins and in the rank order of wins, compared to the results for the three groups of five tournament format. Additionally, there is less variance in the winning percentages among the top eight strategies. Both of these results are due to having more groups, each with a smaller number of entries in the first stage, that allows more of the top strategies to move on to the second and final stage.

## Four-stage Match-up Tournament

The final format considered is a single-elimination, match-up tournament. In this tournament design each entry was paired with one other entry in stage one, where the winner of this stage moved on to stage two. This process continued, with each winner moving on to the next stage, until a final winner was determined in the fourth and final stage. Note that this format works best when the number of entries, $n$, is an integer determined by $n = 2^s$, where $s$ is the number of stages. Because we began with only 15 (rather than 16) entries, we gave each entry a 1 in 15 chance of getting a first-stage "bye". Thus, the fifteen original entries were reduced to eight at the end of stage 1. This design results in over 81 billion different tournament combinations. Rather than examine all possible combinations, we produced a random sample of 100,000 unique tournament combinations. The results of this simulation are summarized in Table S1b. The first column shows the program number and the second column shows the program name. Subsequent columns list the number and percentage of wins in each of the four stages.

Notice that even a relatively weak entry, like RAN, posted a number of first-stage wins. This is due in part to the 1 in 15 chance of receiving a first-stage bye. The results from this match-up design are substantially different from the previous round-robin tournaments. Here programs JO and FE won 58.6% and 23.1%, respectively, of the tournaments played. For JO this is explained as the program beats twelve of the other fourteen entries and ties the remaining two (FR, FE). Thus, in match-up competitions it only loses in tie-break situations. A similar explanation is offered for FE, which beats ten of the other entries, ties two and loses to only one entry, GR. T&C, which performed well in both of the alternative round-robin tournaments, posted the third

highest win percentage (10.1%) in the match-up format. TFT, which does not beat a single strategy (but does tie seven other entries) wins only five of the 100,000 simulated tournaments.

Table S1a. Analysis of Axelrod's First Tournament Using a Two-stage Round-Robin Tournament with Five Groups of Three Entries

| | | Stage 1 Wins | | Stage 2 Wins | |
|---|---|---|---|---|---|
| Program | Name | # | % Wins | # | % Wins |
| 1 | TFT | 33,389 | 33.4% | 15,839 | 15.8% |
| 2 | T&C | 63,360 | 63.4% | 24,277 | 24.3% |
| 3 | NY | 42,204 | 42.2% | 16,775 | 16.8% |
| 4 | GR | 48,673 | 48.7% | 17,556 | 17.6% |
| 5 | SH | 42,084 | 42.1% | 7,570 | 7.6% |
| 6 | S&R | 64,303 | 64.3% | 13,697 | 13.7% |
| 7 | FR | 35,335 | 35.3% | 1,917 | 1.9% |
| 8 | DA | 31,895 | 31.9% | 845 | 0.8% |
| 9 | GR | 40,790 | 40.8% | 847 | 0.8% |
| 10 | DO | 33,105 | 33.1% | 525 | 0.5% |
| 11 | FE | 19,654 | 19.7% | 133 | 0.1% |
| 12 | JO | 12,097 | 12.1% | 19 | 0.0% |
| 13 | TU | 19,872 | 19.9% | - | 0.0% |
| 14 | NA | 6,629 | 6.6% | - | 0.0% |
| 15 | RAN | 6,610 | 6.6% | - | 0.0% |

Table S1b. Analysis of Axelrod's First Tournament Using a Two-stage Round-Robin Tournament with Three Groups of Five Entries

| | | 1st Stage | | 2nd Stage | | 3rd Stage | | Final Stage | |
|---|---|---|---|---|---|---|---|---|---|
| Program | Name | # | p(Win) | # | p(Win) | # | p(Win) | # | p(Win) |
| 1 | TFT | 30,055 | 30.1% | 6,297 | 6.3% | 657 | 0.7% | 5 | 0.0% |
| 2 | T&C | 86,699 | 86.7% | 65,568 | 65.6% | 37,192 | 37.2% | 10,126 | 10.1% |
| 3 | NY | 30,142 | 30.1% | 5,249 | 5.2% | 435 | 0.4% | 4 | 0.0% |
| 4 | GR | 63,270 | 63.3% | 31,073 | 31.1% | 9,975 | 10.0% | 1,194 | 1.2% |
| 5 | SH | 46,695 | 46.7% | 13,628 | 13.6% | 1,763 | 1.8% | 25 | 0.0% |
| 6 | S&R | 76,694 | 76.7% | 48,100 | 48.1% | 20,381 | 20.4% | 2,867 | 2.9% |
| 7 | FR | 63,244 | 63.2% | 31,854 | 31.9% | 12,073 | 12.1% | 4,087 | 4.1% |
| 8 | DA | 50,040 | 50.0% | 15,408 | 15.4% | 1,938 | 1.9% | 23 | 0.0% |
| 9 | GR | 39,886 | 39.9% | 7,694 | 7.7% | 354 | 0.4% | - | 0.0% |
| 10 | DO | 49,995 | 50.0% | 16,496 | 16.5% | 2,655 | 2.7% | 18 | 0.0% |
| 11 | FE | 83,273 | 83.3% | 61,969 | 62.0% | 40,391 | 40.4% | 23,054 | 23.1% |
| 12 | JO | 93,392 | 93.4% | 83,799 | 83.8% | 71,652 | 71.7% | 58,597 | 58.6% |
| 13 | TU | 46,651 | 46.7% | 10,565 | 10.6% | 517 | 0.5% | - | 0.0% |
| 14 | NA | 26,740 | 26.7% | 2,051 | 2.1% | 17 | 0.0% | - | 0.0% |
| 15 | RAN | 13,224 | 13.2% | 249 | 0.2% | - | 0.0% | - | 0.0% |
| | | 800,000 | | 400,000 | | 200,000 | | 100,000 | 100.0% |
